# Parasite-Produced MIF Cytokine: Role in Immune Evasion, Invasion, and Pathogenesis

**DOI:** 10.3389/fimmu.2019.01995

**Published:** 2019-08-21

**Authors:** Swagata Ghosh, Nona Jiang, Laura Farr, Renay Ngobeni, Shannon Moonah

**Affiliations:** ^1^Division of Infectious Diseases and International Health, Department of Medicine, University of Virginia, Charlottesville, VA, United States; ^2^Department of Medicine, Yale University, New Haven, CT, United States; ^3^Department of Environmental, Water, and Earth Sciences, Tshwane University of Technology, Pretoria, South Africa

**Keywords:** MIF, cytokine, protozoan parasites, host-parasite interaction, immune evasion, immunopathology, immunotherapeutic target

## Abstract

Protozoan parasites represent a major threat to health and contribute significantly to morbidity and mortality worldwide, especially in developing countries. This is further compounded by lack of effective vaccines, drug resistance and toxicity associated with current therapies. Multiple protozoans, including *Plasmodium, Entamoeba, Toxoplasma*, and *Leishmania* produce homologs of the cytokine MIF. These parasite MIF homologs are capable of altering the host immune response during infection, and play a role in immune evasion, invasion and pathogenesis. This minireview outlines well-established and emerging literature on the role of parasite MIF homologs in disease, and their potential as targets for therapeutic and preventive interventions.

## Introduction

Protozoan parasites cause more than one million deaths annually. For example*, Plasmodium falciparum*, a protozoan parasite responsible for most human malaria, accounted for an estimated 200 million malaria cases and roughly 500,000 malaria deaths in 2015 (1, 2). The protozoan parasite *Leishmania* causes an estimated 50,000 deaths per annum through visceral leishmaniasis (3). *Entamoeba histolytica* is a protozoan parasite that causes colitis (inflammatory diarrhea). Millions of people are infected with *E. histolytica*, making amebic colitis a leading cause of severe diarrhea, estimated to kill more than 50,000–100,000 people each year (4–6). The protozoan parasite *Toxoplasma gondii*, which affects up to a third of the world's population, is adapted to survive and abide chronically in its host (7). The threats posed by protozoan parasites are further compounded by lack of any effective parasite vaccine, emerging drug resistance, drug toxicity, poor efficacy, and limited antimicrobial options (5, 8, 9). Therefore, identifying novel targets for therapeutic intervention and vaccine prevention is urgently needed. Pathogenic protozoans produce virulence factors that enable immune response evasion and host invasion which promote their transmission and ability to cause human disease (10). Targeting these virulence factors required to cause host damage and disease might successfully treat and prevent these infectious diseases.

The pathogenesis of protozoan diseases is highly variable, and is often influenced by individual life cycles and immunologic consequences of infection. The complicated life cycle of *Plasmodium* begins when an infected female anopheles mosquito injects sporozoites into the bloodstream of a human during a blood meal, which travel to the liver, before emerging to release merozoites into the bloodstream. These merozoites invade and multiply within erythrocytes to rupture, releasing more merozoites, and continually perpetuating invasion by the parasite. *Plasmodium* promotes its survival by avoiding excessive exposure to the immune system by infecting hepatocytes and erythrocytes. Clinical symptoms are associated with the rupture of infected erythrocytes and the release of malarial toxins, and include fever, severe hemolytic anemia and other systemic features. Merozoites also develop into sexual forms known as gametocytes, which are ingested during mosquito bites to continue the life cycle (11). Similarly, *Leishmania* is also a vector-borne protozoan parasite, that is transmitted when *Leishmania* promastigotes are inoculated into the subdermis of the skin by the bite of an infected female phlebotomine sand fly. *Leishmania* is rapidly phagocytized by neutrophils. Promastigotes within dead infected neutrophils are taken up by host macrophages, morphing into the amastigote form. Depending on the species, amastigotes replicate within the macrophage locally to form disfiguring skin ulcers (cutaneous leishmaniasis) or disseminate to the bone marrow, liver, and spleen (visceral leishmaniasis) which is fatal if untreated (12–15).

In contrast to these vector-borne infections, the transmission of the highly prevalent protozoa, *Toxoplasma gondii*, is fecal-oral, through the ingestion of the oocyst from material contaminated with feline feces or undercooked meat infected with tissue cysts. Following intestinal infection, tachyzoites form, and then disseminate to other tissues in the body including the brain, eye, muscle, liver, and placenta. Like *Leishmania*, Toxoplasma is able to infect phagocytes, which facilitates successful infection. Symptoms of primary infection include fever, adenopathy, headache, and myalgia. The stimulation of a robust immune response controls the acute infection, driving the parasite into a chronic, asymptomatic stage allowing *Toxoplasma* to survive as bradyzoites in cyst forms within multiple tissues capable of later reactivation (7). Infection with *E. histolytica* also begins with the ingestion of fecally contaminated food or water, but has a relatively simpler life cycle. *E. histolytica* exists as either infective cysts which are ingested or transforms into invasive trophozoites that penetrate the mucus layer of the large intestine to cause colitis leading to diarrhea, dysentery, and colonic ulceration. The trophozoites can also on occasion disseminate to cause extra-intestinal disease, with a particular predilection for the liver leading to amebic liver abscess (16). Thus, in order to complete their life cycle, all of these protozoa must be able to invade and pass from host to host while avoiding clearance by the immune response. In this minireview, we describe how protozoa secrete a specific protein macrophage migration inhibitory factor to accomplish this task.

## Macrophage Migration Inhibitory Factor

Macrophage migration inhibitory factor (MIF) was one of the first cytokines to be discovered over 50 years ago (17, 18). Since then, a significant amount of information has been accumulated regarding the role of MIF in normal physiology and pathology. MIF is a well-studied pleiotropic inflammatory protein, expressed by a variety of cells, and is a critical upstream mediator of innate immunity. While MIF's exact molecular mechanism is not fully understood, partial pathways of MIF signaling have been established. For example, secreted MIF binds to its receptor, CD74, on immune cells, activates the ERK1/2 and PI3K/Akt pathways, and modulates expression of various cytokines, e.g., TNF-α, IL-6, IL-8, and IL-12 (19). MIF may also bind to CXCR2 and CXCR4, which may be responsible for its chemotactic properties. In addition, MIF stimulates the production of matrix metalloproteinases (20). Therefore, it is not surprising that MIF plays an important role in immunity and that excess MIF expression has been linked to exaggerated inflammation and immunopathology in diseases such as rheumatoid arthritis, and inflammatory bowel disease (19, 21, 22).

The proinflammatory properties of MIF also make it a crucial mediator in the immune response against a wide variety of pathogens including parasites (23). In protozoan infection, host MIF play a key role in reducing parasite burden through stimulation of both innate and adaptive immune cells. Mechanistically, host MIF can stimulate nitric oxide production by macrophages and dendritic cells, which in turn eliminates parasites such as *Leishmania, Toxoplasma*, and *Trypanosoma* (13, 23, 24). MIF can also be harmful to the host. That is, MIF production has been linked to pathology during malaria and *T. brucei* infection, by promoting inflammation-induced tissue damage (21, 25, 26). The role of host MIF during parasite infections has been well-reviewed elsewhere (23, 24, 27).

**Figure 1:**
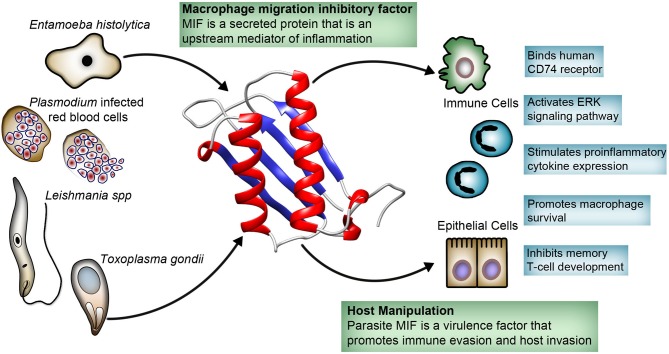
Host-parasite interaction involving protozoa-produced macrophage migration inhibitory factor (MIF). Protozoa secrete MIF that is structurally similar to human MIF. Protozoa MIF binds directly to the human MIF receptor CD74, activating the ERK pathway with immunomodulatory effects on variety of immune and epithelial cells. Protozoa MIF immunomodulatory effects appear to play a role in parasite invasion and immune evasion, and has been linked to pathogenesis.

Counterintuitively, many pathogenic protozoans, including *Plasmodium, Entamoeba, Toxoplasma*, and *Leishmania*, produce their own MIF cytokine. These secreted parasite-produced MIF are structurally similar to human MIF, bind the MIF receptor (CD74), and stimulate immune cells and epithelial cells to cause the release of cytokines such as TNF-α, IL-8, and IL-12 (28–35). While it seems counterintuitive for protozoans to secrete a proinflammatory cytokine, it appears they have an important role in the parasite life cycle. Here, we focus on MIF produced by medically important protozoans, highlighting the recent contributions that have improved our understanding of the role of protozoan MIF in immune evasion, invasion, and pathogenesis (Figure 1).

## Immune Evasion

The host deploys a robust immune response to prevent parasite invasion, clear the infectious pathogen, and prevent re-infection. However, parasites have developed a remarkable number of mechanisms to evade these attacks (10). For example, *Leishmania* has developed ways to modify host cell signaling pathways, in order to survive and persist in host cells. *Leishmania* targets macrophages, which, interestingly, are the primary immune cells involved in the parasite's eradication (13). *Leishmania major* encodes two isoforms of MIF which facilitates its persistence in macrophages and contributes to its evasion from immune clearance. *L. major* MIF binds to CD74 on infected macrophages, activating the ERK1/2 pathway and preventing apoptosis of macrophages (35, 36). Infected macrophages then survive a sufficiently long enough time for the parasite to avoid excessive exposure to the immune system and complete its infectious life cycle.

The lack of protective immunity against re-infection is one of the biggest problems in controlling the transmission of *p*rotozoan infections. An adequate amount of protective memory T-cells are needed to fight off re-infection (37). Recent research in parasite MIF has provided a mechanism by which parasites evade the immune response by interfering with the development of immunological memory during infection, allowing them to re-infect their host (28, 29, 35). Using mouse models, researchers found that the proinflammatory effects of both *Plasmodium* and *Leishmania* MIF can manipulate T-cell differentiation. *Plasmodium* MIF enhances the production of IFN-γ and IL-12 which reduces the anti-*Plasmodium* blood-stage CD4 T-cell response. Mice infected with MIF-deficient *P. berghei* had reduced levels of these cytokines. This reduced inflammatory state correlated with improved survival of CD4 T helper cells. As a result, mice were able to develop effective T-cell memory when infected with MIF-deficient parasites which provided a protective response against a subsequent *P. berghei* infection. *Leishmania* MIF cause T-cells to develop into exhausted PD-1^+^ short-lived effector cells with reduced IL-7R expression, which is needed to produce and maintain memory cells (28, 29, 35). These short-lived cells die during infection, and the long-lived memory T-cells required to prevent re-infection were not produced in adequate amounts (29). This MIF-induced lack of memory cells resulted in parasitic re-infection.

**Figure 2:**
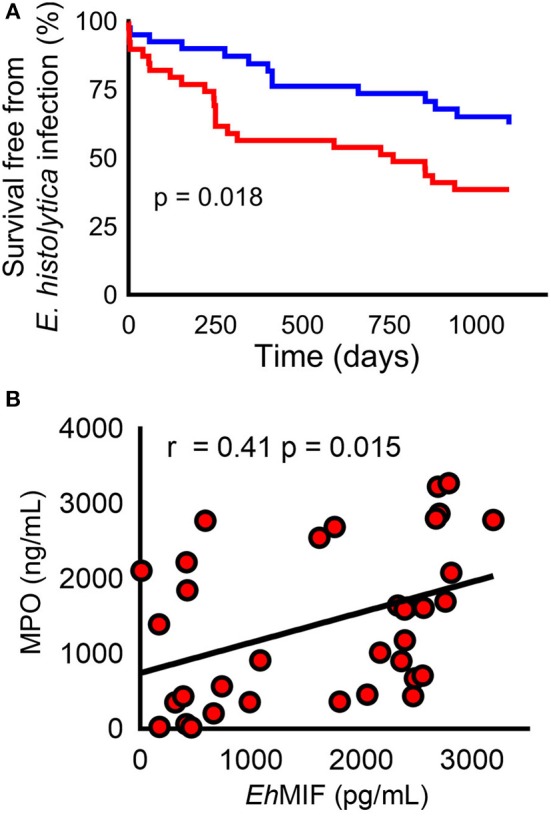
*E. histolyica* MIF (*Eh*MIF) in human amebiasis. **(A)** Children in the top 50th percentile for anti- *Eh*MIF antibody (blue line) had a significantly higher probability of survival free of *E. histolytica* infection than children within lower 50th percentile (red line). **(B)** Significant positive correlation between fecal *Eh*MIF levels and the myeloperoxidase (MPO) marker of intestinal inflammation in persons with amebiasis (*n* = 35). Panels are reproduced from (17) with permission.

Recent clinical observations also support these findings. It was observed that in a cohort of children in an area endemic for amebiasis, those who lacked adequate amounts of antibodies against *E. histolytica* MIF were not protected from future infection (Figure 2A). The authors postulated that *E. histolytica* MIF might share similar properties to *Plasmodium* and *Leishmania* MIF. That is, *E. histolytica* MIF might also inhibit the development of sufficient amounts of memory cells. Thus, antibodies against *E. histolytica* MIF would block this effect resulting in adequate amounts of memory cells to protect against reinfection (32). Nevertheless, further studies are needed to confirm this theory. Also, the role of *Toxoplasma* MIF in immune evasion remains largely understudied.

## Invasion

Host tissue invasion by extracellular or intracellular protozoan parasites play an important role in the pathogenesis of disease. The extent of tissue invasion by extracellular parasites correlates with the degree of disease severity (16). For example, the depth of tissue invasion is associated with worse outcomes in clinicopathological studies of patients with severe amebic colitis (38, 39). On the other hand, obligate intracellular parasites invade host cells to complete their life cycle (13, 40). Several parasite factors are known to contribute to the invasion process. Recent studies have implicated parasite MIF proteins in facilitating invasion and dissemination of several protozoan parasites.

The extracellular matrix (ECM) is a network of proteins that provides tissue support and represents a major physical barrier to the parasite invasion. Matrix metalloproteinases (MMPs) are enzymes primarily responsible for ECM breakdown (41). Protozoa infections trigger an inflammatory response which leads to MMP overexpression, resulting in ECM breakdown. ECM degradation facilitates cell movement, and allows immune cell infiltration at the site of infection for host defense (42). Parasites have developed mechanisms to exploit the activities of MMPs to promote their invasion. For example, MMPs play a critical role in *E. histolytica* invasion. MMP expression is increased in human amebic colitis and inhibition of MMP prevented *E. histolytica* invasion in a human colonic explant model (32, 43, 44). Recently, a causal relationship between *E. histolytica*-produced MIF and gut inflammation was established using cellular and mouse models of amebic colitis. In the same study, researchers found that *E. histolytica* MIF-induced inflammation resulted in increased MMP production (32). Therefore, *E. histolytica* parasites appear to produce MIF as a virulence factor to exploit the inflammatory response to promote tissue invasion.

As mentioned above, neutrophils are the first immune cells to reach the site of *Leishmania* infection after a sand fly bite, and their uptake by neutrophils followed by macrophage engulfment contributes to leishmanial parasites infectivity and assist in life cycle progression (12–14). Neutrophils are short-lived phagocytes that act as a “Trojan horse” used by *Leishmania* parasites to obtain entry into macrophages thereby avoiding cell activation (15). Whether there are leishmanial factors that drive this neutrophil infiltration remains largely unanswered. While host MIF exhibits chemokine-like activities through interactions with the chemokine receptors CXCR2 and CXCR4 (45, 46), protozoan MIF-CXCR2 and CXCR4 interactions remain unclear.

Similar to *Leishmania, T. gondii* induces immune cell infiltration and not only evades their killing, but also hitches a ride in these cells to spread infection (47, 48). *T. gondii* must cross the intestinal barrier for it to advance from the gut to sites of secondary infection, and tachyzoites are often found in neutrophils in the gut lumen. *In vitro* studies have indicated that *T. gondii* MIF stimulates the production of the potent chemoattractant IL-8 from human cells. In an attempt to explain why this would benefit the parasite, it has been suggested that MIF-induced IL-8 production leads to neutrophil recruitment. Infected neutrophils, which are incapable of clearing the parasite, serve as motile reservoirs for *T. gondii* infection, facilitating the transepithelial migration of the parasite (30, 48, 49). While plausible, additional studies are warranted to validate the role of *Toxoplasma* MIF in invasion.

## Pathogenesis

During protozoan infections, an unbalanced inflammatory reaction increases tissue destruction which leads to clinical disease. The inflammatory response is essential in that it provides protection against invading microbes. However, protozoan parasites have developed effective strategies to evade the immune response, avoid elimination, and persist in their host, which exacerbates the damage caused by the lingering inflammatory response to invading parasites (10). This is further compounded by the fact that these parasites secrete MIF cytokine that can directly drive inflammation.

Host cytokines released during *Plasmodium* infection contribute to severe malaria. For example, high TNF-α production is a strong predictor of severe malarial anemia and cerebral malaria in children (50, 51). *P. falciparum* MIF was shown to stimulate TNF-α secretion by immune cells *in-vitro*. Also, circulating serum *P. falciparum* MIF levels positively correlated with serum TNF-α levels in malaria patients, and higher *P. falciparum* MIF levels were observed in patients with severe malarial anemia and cerebral malaria (25, 28). These findings suggest that *P. falciparum* MIF is likely contributing to immunopathogenesis during malaria.

Neutrophil infiltration is a hallmark of amebic colitis. Neutrophils generate oxygen free radicals that are capable of killing the *E. histolyica* parasite. That said, *E. histolytica* has developed several strategies to counter and survive neutrophil killing (52–54). This results in an excessive and persistent neutrophil response in the gut that has been shown to be associated with the most severe forms of human amebiasis, which also carry high fatality (6, 55–57). *E. histolyica* MIF plays an essential role in neutrophil infiltration during infection. *E. histolytica* MIF was shown to stimulate IL-8 and the murine IL-8 homolog KC (potent neutrophil chemoattractants), resulting in neutrophil infiltration and tissue destruction in cellular and mouse models. A recent human study found that gut *E. histolyica* MIF levels correlated with intestinal inflammation severity [Figure 2B; (32, 33)].

Macrophages also play a crucial role in protozoan MIF-induced immunopathology. *In vitro* studies show that *E. histolyica* MIF directly enhances TNF-α and IL-6 production from macrophages (31). Both cytokines cause collateral tissue injury in amebic colitis and liver abscess (10, 58). In a mouse model of *Leishmania* infection, *Leishmania* MIF upregulated inflammatory and innate immune signaling in infected macrophages, such as CXCL1, TLR2, and TNF-α, when compared to *MIF*^−/−^ strains (35). Taken together, this pro-inflammatory phenotype, extending the survival of infected macrophages, and defective adaptive immune response supports the contribution of *Leishmania* MIF to the chronic destructive inflammatory state observed in leishmaniasis.

## Other Protozoans Producing MIF

Other medically important protozoans include *Trichomonas, Giardia, Trypanosoma, Acanthamoeba*, and *Naegleria*. MIF orthologs have been discovered in *Trichomonas* and *Giardia*. The structure of *Giardia* MIF has been solved with a characterization similar to human MIF, but its role in infection is not well-understood (59). Surprisingly, MIF orthologs have not been characterized in *Trypanosoma, Acanthamoeba*, and *Naegleria*. However, incomplete genome assembly and annotation may limit *in-silico* analysis and explain why MIF has yet to be identified in these protozoans.

Inflammation is a critical component of tumor progression and many cancers, including prostate cancer, arise from sites of infection and chronic inflammation (60, 61). *Trichomonas vaginalis* is a sexually transmitted parasite that can colonize the prostate in men. *T. vaginalis* also secretes MIF which has pro-inflammatory properties. In addition to stimulating the production of IL-8 and IL-6 cytokines, *Trichomonas* MIF binds to the human CD74 MIF receptor triggering the activation of the pro-proliferative ERK and P13K/Akt pathways in prostate epithelial cells. *Trichomonas* MIF-driven inflammation and cell proliferation, was linked to the promotion and progression of prostate cancer (62).

## Conclusion

Recent studies have made it increasingly clear that parasite-produced MIF is a virulence factor that play a significant role in host-parasite interactions and contributes to pathogenesis. Despite these advances, key questions remain unanswered. Such as, can we translate these findings to provide beneficial interventions to patients infected with these pathogens? Do we know enough to intervene in a meaningful way? Protozoan MIF (P-MIF) appears to be a logical candidate for further evaluation as an effective immunotherapeutic target given the accumulation of data showing that: (i) infected persons naturally make antibodies against P-MIF, (ii) anti-P-MIF do not cross-react with host MIF, and (iii) neutralizing antibodies inhibit P-MIF activity and therefore prevent re-infection and reduce immunopathology.

## Author Contributions

SG, NJ, LF, RN, and SM wrote different sections, edited, and reviewed the manuscript.

### Conflict of Interest Statement

The authors declare that the research was conducted in the absence of any commercial or financial relationships that could be construed as a potential conflict of interest.
